# Enhancing earthquake preparedness knowledge and practice among Nepalese immigrants residing in Japan

**DOI:** 10.1038/s41598-023-31729-y

**Published:** 2023-03-18

**Authors:** Aliza K. C. Bhandari, Mahbubur Rahman, Osamu Takahashi

**Affiliations:** 1 Graduate School of Public Health, St. Luke’s International University, Tokyo, Japan; 2 Department of Health Policy, National Center for Child Health and Development, Tokyo, Japan; 3 OMURA Susumu & Mieko Memorial, St. Luke’s Center for Clinical Academia, 5Th Floor, 3-6-2, Tsukiji, Chuo-Ku, Tokyo, 104-0045 Japan

**Keywords:** Natural hazards, Health care

## Abstract

This study aims at increasing earthquake preparedness knowledge perception and practice among Nepalese immigrants residing in Japan through an educational intervention. A single arm quasi experimental study was conducted among Nepalese immigrants residing in Japan. An educational intervention was prepared along with a 52 itemed questionnaire. In total, 165 participants responded to our questionnaire. Majority of them were male (67.88%), and the mean age was 32.78 years. Generalized equation model showed that the knowledge score of earthquake preparedness was 4.01 points higher immediately after the intervention [95% CI (2.78–5.24), p-value < 0.001] compared to baseline with a further increase by 7.02 points [95% CI (5.96–8.09), p-value < 0.001] at two weeks follow up. However, the practice score increased only by 2.83 points [95% CI (2.51–3.14), p-value < 0.001] immediately after the intervention with a similar increase at two weeks and 12 weeks follow up period [OR: 2.62, 95% CI (2.29–2.96), p-value < 0.001]. The educational intervention, when conducted in native language, can increase both the knowledge and practice score of earthquake preparedness hence, information related to earthquake preparedness in Nepali languages in the government websites of Japan could potentially increase information seeking behavior of people.

## Introduction

Disaster preparedness refers to a process or act of collecting necessary information, ensuring materials and resources required to mitigate or minimize the effect of a disaster^1^. Preparedness of a disaster depends on the type of disaster and the health seeking behavior of an individual, hence those who are aware of the effects of a disaster tend to prepare early to prevent the adverse effects of a disaster^2^. The world has seen thousands of disasters till today however, most people are not prepared for the disaster until the misery happens^3^. Therefore, it is crucial to identify some effective interventions in promoting the knowledge and practice of disaster preparedness^4^.

Earthquakes are one of the major disasters that has caused the most harm throughout the world in the last few decades^5^. Earthquakes in Japan are quite frequent with about nine earthquakes of more than or equal to five Japan Meteorological Agency (JMA) magnitude felt only in the month of May 2022 which affected about hundreds of people^6^. “The Great Kanto earthquake”, “The Great Hanshin earthquake”, and “The Great East Japan earthquake” occurred in the last several decades are still considered as few of the major disasters that mankind had experienced till date^7–9^. There are several governmental and non-governmental organizations which have been working for disaster risk reduction with an active involvement of the community, many disaster risk reduction programs have been implemented. However, the sustainability of such programs has raised several concerns^10,11^.

Nevertheless, there is evidence which suggests that preparing for any kind of disaster or emergency situation on a day-to-day basis could overcome the existing challenges in disaster management^12^. And there are multiple factors which could determine all kinds of disaster preparedness and not limited to earthquake preparedness like socioeconomic differences, language barriers, races and ethnicity, previous exposure to the disasters, etc.^13–15^. Japan is one of the countries with robust disaster management policies^16^. However, the efforts of the Japanese government towards disaster management among the immigrant population is barely known^17^. Nepalese are the sixth largest immigrant group of Japan. Several studies have already identified the barriers in accessing health care among them. Similarly, it has already been identified that Nepalese immigrants residing in Japan have poor knowledge and practice regarding disaster preparedness. The major barriers identified by immigrants in accessing knowledge, attitude and practice regarding natural disasters were language followed by the lack of information^18,19^. Having said that, only a limited number of Japanese official prefectural government websites provide their information in Nepali language. Nepalese residing in Japan are mostly blue-collar workers with average educational level and low Japanese language ability^20^. Many of them are not aware of the disaster preparedness drills that the Japan government provides in several cities of Japan. There are limited number of studies conducted in earthquake preparedness, among them studies conducted in other countries such as the USA and Iran, along with Japan have shown that the level of earthquake preparedness varies between the natural inhabitants of the society and the immigrant population^21–24^. Similarly, several myths exists among people when it comes to taking immediate action during an earthquake like standing in a doorway, running out from home in the fear of building collapse, etc.^25^. Hence, it is crucial to enhance the earthquake preparedness skills among the vulnerable group of population by understanding their beliefs and risk perceptions^26^.

Disaster drills and practices have been proven to mitigate the risk of various health morbidities and mortalities during a disaster situation^27^. Moreover, if such drills and practices are focused to meet the local needs of people then these are more likely to have a higher impact^28,29^. Similarly, intervention focusing only on providing information related to hazard or risk has not been proven to enhance the practice of disaster preparedness, however, interventions when aggregated with trainings and two-way interactions among participants and the interventionist have shown some better results^26,30^. Identifying and providing training to the leaders who could possibly disseminate the relevant information to their local community is also one of the possible ways in increasing the preparedness level of individuals^31^. The earthquake preparedness intervention developed so far in Japan are limited and are focused upon the Japanese population and their level of understanding whereas the preparedness programs in low-income countries like Nepal are mostly focused on medical professionals and not on the individual level of preparedness^32^. Thus, this study aims at enhancing the earthquake preparedness knowledge and practice solely among Nepalese immigrants residing in Japan through an educational intervention developed in their native language with extensive information on earthquakes and majors to follow before, during and after the earthquakes while providing necessary contact information and introducing some reliable source of information that people could access during earthquakes.

## Methods

### Study design and population

A single arm quasi experimental study was conducted using a structured questionnaire to identify the change in knowledge perception and practice of earthquake preparedness among Nepalese immigrants residing in Japan before, immediately after, 2 weeks after and 12 weeks after the intervention respectively. We included Nepalese immigrants with more than three months’ residence permit aged 18 years or above in this study. A web-based survey was used to collect information from the participants after they provided written informed consent to participate in the study from November 20th, 2021, to March 30th, 2022.

### Development and validation of intervention

An educational intervention was developed by in-depth literature review, and information on earthquake preparedness was also obtained from the Tokyo Metropolitan Government websites on disaster prevention and management^33–35^. The collected information was translated in Nepali language by the native speaker (AKB). The Microsoft PowerPoint slides were prepared with contents like description of intervention, aims and objectives of the research, introduction to earthquake, things to consider before earthquake, demonstration on emergency bag preparation, what should be done during earthquake, what should not be done during earthquake, how to do drop, cover and hold during earthquake, what are the signs used for disaster situations, emergency contact numbers, some important websites and twitter accounts to get information on earthquake and so on. Lecture, discussion, and demonstration methods were used to deliver the intervention. The materials were prepared so that the lecture and discussion part would take about 45 min, the demonstration would take 10 min of time and 5 min was left for the question-and-answer session.

The intervention was back translated into English and was sent to language experts for checking any inconsistencies. Meanwhile, we conducted a focus group discussion to validate the intervention. We provided intervention to two different groups of people (six members in each group). We asked them for any suggestions to improve the intervention and with some minor revisions the intervention was finalized.

### Development and validation of the questionnaire

At first a 61 itemed questionnaire was created through in-depth literature review to assess the knowledge perception and practice regarding earthquake preparedness among Nepalese immigrants residing in Japan. The tool was then translated into Nepali language by the native speaker AKB. We then performed psychometric validation of the questionnaire by analyzing content validity, construct validity, reliability and performing exploratory factor analysis^36^.

A panel of seven Nepali experts from the United States (n = 1), Nepal (n = 3) and Japan (n = 3), who had substantial contributions to the field of disaster management was formed. They were asked to review the 61 itemed questionnaire and provide their responses in a four-point Likert scale ranging from “Highly relevant” to “Not relevant”^37,38^. Experts were asked to choose “Highly relevant” if they think the items were relevant and applicable to Nepalese immigrants residing in Japan, whereas they were requested to score “Quite relevant” if the items were relevant but needed slight modifications. Similarly, they were asked to score “Somewhat relevant” if the items needed substantial modification and “Not relevant” if the items were not necessary in the questionnaire. We calculated the scale-based and item-based content validity index (i-CVI) based on our experts’ opinion and 50 items were retained (i-CVI > 80%) after reducing 13 items (i-CVI and s-CVI < 80%)^39^, we added two more items and modified four items as per the experts’ opinion.

After completing this procedure, we used this tool during our two focus group discussions among the representative pilot groups with six members each. We asked the participants if some items were difficult to understand or not appropriate for Nepalese society or are completely unrelated to our intervention. We calculated the interrater agreement using modified Kappa statistics and since all items had a Kappa value of more than or equals to 0.74 we finalized the three-part questionnaire with 52 items in total^40^. The first section of the questionnaire consisted of 23 items to assess general knowledge perception and practice of earthquake preparedness in a 3-point Likert scale with “Yes”, “No” and “Don’t know/Not applicable” option where “Don’t know/ Not applicable was scored as 0 and scored as 1 if the participants provided correct responses to the questions in terms of “No” or “Yes”. Hence, the maximum score a participant could get from the tool was 23 and the minimum was zero. The questions were quite general in this section like “Have you experienced earthquake?”, “Do you think your building is more vulnerable to earthquake damage?”, “Do you know the evacuation center near your home?”, “Have you seen a building collapse hazard map for your area?”, “Do you know where you should keep your emergency bag?”, “Do you know how a disaster shelter works during major disasters?” and so on.

Similarly, the second part consisted of 15 items to assess the effectiveness of the educational intervention regarding earthquake preparedness. Participants were asked to choose “True” if the information provided was accurate and “False” if the information provided was not accurate based on their own perception. They were given one point for correct response and zero for the incorrect response hence the total score they could obtain in this section was 15. The True/False questions covered basic facts like “in an earthquake they should get down close to the ground” or “one should get under a big piece of furniture such as desk or other covers”, “one should be standing near the doorways in an earthquake”, “helping yourself is the priority during earthquake” and so on. The response of people from this section could hence provide an insight on the understanding of the intervention by the participants like if they were able to understand our intervention, they would provide correct responses to these questions.

Fourteen remaining items contained socio-demographic questions including name, year of birth, sex, educational status, area of residence in Nepal and in Japan, type of house living in Japan, period of stay in Japan, contact details with phone number and email address (optional). We also included questions like having formal education in Japan as a proxy to Japanese language ability and working in a Japanese company or not instead of directly asking the household income as per our experts’ suggestions. The 52 itemed questionnaires were sent for back translation into English language to an expert and the Nepali version was further sent to another language expert for any discrepancies.

A pilot study was conducted using the 52-item questionnaire in the 10% of the sample size (n = 20). The Kaiser–Meyer–Olkin (KMO) value was 0.77 and p-value was less than 0.05 in the Bartlett’s Test of Sphericity indicating moderate sample adequacy for the explanatory factor analysis (EFA)^41,42^. The Cronbach’s coefficient alpha indicated high internal consistency (0.92) and EFA (only items included in the first section of the questionnaire were subjected to EFA) using tetrachoric correlation matrix with principal components method for binary variables and varimax rotation resulted in retention of six factors with eigen value more than 1 which explained 65.2% of the variance with 19 items having factor loadings more than 0.50^43,44^. However, all 52 items were included in the survey as they were reported to be highly relevant in Nepalese context by our panel of experts. We then collected data using a 52-itemed questionnaire among larger sample size (N = 165).

### Sample

The sample size was calculated based on our repeated measurement with pre-post study design with one pre-intervention measurement and three post-intervention measurements^45^.

$$ {\text{N}} = {\text{R}} \times \left[ {\frac{{\left( {1 + \varphi } \right)^{2} }}{\varphi }\frac{{(Z_{{1 - \alpha /2}}  + Z_{{1 - \beta }} )^{2} }}{{\left( {\delta _{{Plan}} /\sigma _{{Plan}} } \right)^{2} }} + \frac{{Z_{{1 - \alpha /2}}^{2} }}{2}} \right], $$

$$ {\text{R}} = \left[ {\frac{{1 + \left( {w - 1} \right)\rho }}{w} - \frac{{v\rho ^{2} }}{{\left[ {1 + \left( {v - 1} \right)\rho } \right]}}} \right] $$

where, δ_*plan*_ = mean effect size (40% more than the standard population)^15^, σ_*plan*_ is the corresponding standard deviation, φ is the 1 as it is pre-post study design and the ratio between pre-test and post-test sample is 1:1, Z_1−α/2_ is the 1.96 (α = 0.05), Z_1−β_ is the 1.28 (β = 0.1), V is the number of measurements before intervention; it is 1 in this study, W is the number of measurements after the intervention; it is 3 in this study, R is the design effect, ρ is the intra-class correlation coefficient; we considered 0.5 as our data are highly correlated.

Hence, the sample size was estimated as 149 to detect 3 points in difference of knowledge or practice score (with a standard deviation of 6)^15^ between pre-test and post-test observations.

### Informed consent and data collection

A web-based written informed consent was obtained from the participants for their participation in this study after the complete disclosure of research objectives and intervention, participation criteria, pros and cons of participation, data sharing and privacy disclosure information. Participants were requested to provide their name, contact details, type of visa status, age, and email address so that they could be contacted during the day of intervention and be reminded of the follow up periods. Among all participants providing informed consent only participants aged 18 years or more and having residency status with more than three months residence permit were contacted by AKB via direct calls, emails, or social networking sites (Facebook’s messenger application) and the information on the Zoom link for the day of intervention were provided via channels requested by them. Participants were divided into five different groups to accommodate their availability and the intervention was provided via Zoom on every weekend or public holidays starting from November 2021 to December 2021 hence, the 12 weeks follow up of the last group ended in March 2022. The questionnaire was distributed using the google form link via Zoom online. The follow up surveys were conducted immediately, at 2 weeks and 12 weeks after the intervention.

### Data analysis

Frequency, mean, and standard deviation were calculated. Paired t-test was used to identify the mean change in knowledge and practice scores during the postintervention periods compared to that of the preintervention period. Generalized Estimating Equation (GEE) model was used to find the efficacy of intervention at various time points and factors associated with the change in knowledge and practice score as we were interested in population average rather than variance at different levels and the coefficient estimates obtained by the generalized estimating equations (GEE) typically used to estimate population average models (sometimes called marginal models) which describe changes in the population mean given changes in covariates. We used autoregressive correlation structure as we assumed progressively lower correlation between successive measurements and used the gaussian family with link identity for the GEE model^46^. All data coding and analyses were conducted using Stata 16.1.

### Ethical approval and consent to participate

This study got ethical approval from the Ethical Review Board of St. Luke’s International University, Japan after getting reviewed the research proposal with the International Review Board (IRB) approval number “21-E001”. We obtained written informed consent from the participants after the disclosure of the objectives, research methodology, data sharing and utilization policy of this research, anticipated risks, burdens and benefits and institutional affiliations of the researcher for this study. We also collected data on personal identifying variables like name, age, year of birth, address, email, contact number on complete disclosure of data sharing and utilization policy of this research. The participants were not involved in the design or conduct or reporting or dissemination plans of this research. The intervention and all methods were performed in accordance with relevant guidelines and regulations.

## Results

A total of 165 participants completed the questionnaire with mean age of 32.78 ± 8.70, and most of them were male (67.90%). More than half of the participants had a bachelor’s degree or higher, were heavily concentrated in the Kanto region of Japan (55.76%) and had some formal education in Japan (57.58%). Majority (> 60%) of the respondents had been residing in Japan for more than 5 years and working in Japanese companies. About 1/3rd of the participants was living in concrete houses and more than 60% were living in a neighborhood with many Nepalese immigrants. The response rate was 95.20%, 92.10% and 89.10% immediately after the intervention, two weeks after the intervention and 12 weeks after the intervention respectively. We performed Little’s MCAR test and found that the variables were missing completely at random (p-value > 0.05). The drop-out rates were significantly different among male and female and among various educational categories while comparing the baseline and the 12 weeks follow up survey using Fisher’s exact test (Table 1) however, we only analyzed non-missing observations in our GEE model in Tables 2 and 3.

**Table 1:** Sociodemographic distribution of study participants at baseline and dropout rates at the 12 weeks follow up period.

	Frequency (percentage)		p-value
	Before intervention (N = 165)	Dropouts at 12 weeks f/o after intervention (N = 18)	
Age group			0.302
18–25	34 (20.61)	4 (11.76)	
26–35	74 (44.85)	8 (10.81)	
36–45	37 (22.42)	6 (16.22)	
46 or more	20 (12.12)	0 (0.00)	
Sex			0.033*
Male	112 (67.88)	16 (14.29)	
Female	53 (32.12)	2 (3.77)	
Highest education			0.044*
SEE or less	18 (10.91)	1 (5.56)	
High school	44 (26.67)	4 (9.09)	
Bachelors or equivalent	84 (50.91)	7 (8.33)	
Masters or above	19 (11.52)	6 (31.58)	
Province in Nepal			0.454
Province 1	13 (7.88)	3 (23.08)	
Province 2	12 (7.27)	1 (8.33)	
Bagmati Province	58 (35.15)	7 (12.07)	
Gandaki Province	43 (26.06)	2 (4.65)	
Lumbini Province	20 (12.12)	3 (15.00)	
Karnali Province	13 (7.88)	2 (15.38)	
Sudurpaschim Province	6 (3.64)	0 (0.00)	
Region in Japan			0.412
Hokkaido	1 (0.61)	0 (0.00)	
Tohoku	14 (8.48)	0 (0.00)	
Kanto	92 (55.76)	11 (11.96)	
Chubu	9 (5.45)	0 (0.00)	
Kansai	5 (3.03)	0 (0.00)	
Chugoku	2 (1.21)	0 (0.00)	
Shikoku	2 (1.21)	1 (50.00)	
Kyushu	40 (24.24)	6 (15.00)	
Period of stay in Japan (in years)			0.645
0–4	60 (36.36)	5 (8.33)	
5–9	74 (44.85)	10 (13.51)	
≥ 10	31 (18.79)	3 (9.68)	
Have taken formal education in Japan			0.523
Yes	95 (57.58)	10 (10.53)	
No	70 (42.42)	8 (11.43)	
Working in a Japanese company			0.545
Yes	94 (56.97)	10 (10.64)	
No	71 (43.03)	8 (11.27)	
Type of housing			0.074
Weak	46 (27.88)	2 (4.35)	
Concrete	119 (72.12)	16 (13.45)	
Living in a neighborhood with many Nepalese immigrants			0.369
Yes	102 (61.82)	10 (9.80)	
No	63 (38.18)	8 (12.70)	

p-value: level of significance among those who dropped out of the intervention at 12 weeks follow up period and those who didn’t obtained by Fisher’s exact test, *p-value < 0.05, N: Total sample size, f/o: follow up, SD: Standard Deviation, SEE: Secondary Education Examination (refers to the examination of 10th grade of schooling in Nepal), dropout rate = number of dropouts at 12-weeks post-intervention follow-up/total number of respondents at pre-intervention × 100.

**Table 2:** Generalized estimating equation model for earthquake preparedness knowledge among Nepalese immigrants residing in Japan (N = 165).

	β (95% CI)	SE	p-value
Time of intervention			
Before intervention	1		
Immediately after intervention	4.01 (2.78–5.24)	0.63	< 0.001***
After two weeks of intervention	7.02 (5.96–8.09)	0.54	< 0.001***
After 12 weeks of intervention	5.96 (4.78–7.14)	0.60	< 0.001***
Age	0.01 (− 0.03 to 0.05)	0.02	0.709
Sex			
Male	1		
Female	− 0.26 (− 1.02 to 0.50)	0.39	0.507
Highest education			
SEE or less	1		
High school	0.57 (− 0.51 to 1.65)	0.55	0.304
Bachelors or equivalent	0.65 (− 0.59 to 1.90)	0.64	0.303
Masters or above	0.40 (− 1.00 to 1.81)	0.72	0.573
Province of Nepal			
Province 1	1		
Province 2	− 0.82 (− 2.41 to 0.77)	0.81	0.312
Bagmati Province	− 0.29 (− 1.53 to 0.96)	0.63	0.653
Gandaki Province	− 0.14 (− 1.64 to 1.36)	0.76	0.857
Lumbini Province	− 0.63 (− 2.22 to 0.96)	0.81	0.439
Karnali Province	0.24 (− 1.44 to 1.91)	0.85	0.782
Sudurpaschim Province	1.26 (− 0.48 to 3.00)	0.89	0.157
Region			
Kanto	1		
Hokkaido	0.05 (− 1.37 to 1.47)	0.72	0.947
Tohoku	0.19 (− 1.03 to 1.40)	0.62	0.763
Chubu	− 0.04 (− 2.30 to 2.21)	1.15	0.969
Kansai	− 0.23 (− 2.80 to 2.34)	1.31	0.861
Chugoku	− 1.05 (− 3.24 to 1.13)	1.12	0.345
Shikoku	− 2.06 (− 7.69 to 3.56)	2.87	0.472
Kyushu	− 0.77 (− 1.63 to 0.10)	0.44	0.084
Period of stay in Japan (in years)			
0–4	1		
5–9	− 0.28 (− 1.13 to 0.56)	0.43	0.511
≥ 10	0.04 (− 1.05 to 1.14)	0.56	0.936
Formal education in Japan			
Yes	1		
No	− 0.60 (− 1.39 to 0.18)	0.40	0.131
Working in a Japanese company			
Yes	1		
No	0.00 (− 0.75 to 0.75)	0.38	0.991
Type of housing			
Weak	1		
Concrete	0.10 (− 0.77 to 0.96)	0.44	0.826

*SEE* Secondary education Examination, *SE* robust standard error, *CI* confidence interval.

***p-value < 0.001.

**Table 3:** Generalized estimating equation model for earthquake preparedness practice among Nepalese immigrants residing in Japan (N = 165).

	β (95% CI)	SE	p-value
Time of intervention			
Before intervention	1		
Immediately after intervention	2.83 (2.51–3.14)	0.16	< 0.001***
After 2 weeks of intervention	2.49 (2.12–2.86)	0.19	< 0.001***
After 12 weeks of intervention	2.62 (2.29–2.96)	0.17	< 0.001***
Age	− 0.01 (− 0.02 to 0.01)	0.01	0.218
Sex			
Male	1		
Female	0.06 (− 0.22 to 0.34)	0.14	0.679
Highest education			
SEE or less	1		
High school	0.33 (− 0.18 to 0.83)	0.26	0.202
Bachelors or equivalent	0.17 (− 0.32 to 0.65)	0.25	0.502
Masters or above	0.26 (− 0.30 to 0.82)	0.29	0.368
Province of Nepal			
Province 1	1		
Province 2	0.11 (− 0.39 to 0.61)	0.26	0.675
Bagmati Province	− 0.44 (− 0.80 to − 0.09)	0.18	0.015*
Gandaki Province	− 0.39 (− 0.79 to 0.00)	0.20	0.053
Lumbini Province	− 0.31 (− 0.74 to 0.12)	0.22	0.163
Karnali Province	− 0.34 (− 0.80 to 0.13)	0.24	0.157
Sudurpaschim Province	− 0.74 (− 1.50 to 0.01)	0.38	0.053
Region			
Kanto	1		
Hokkaido	− 0.09 (− 0.69 to 0.51)	0.31	0.765
Tohoku	0.42 (0.08–0.76)	0.17	0.015*
Chubu	0.57 (0.07–1.08)	0.26	0.027*
Kansai	0.76 (0.19–1.34)	0.29	0.010*
Chugoku	− 0.58 (− 1.22 to 0.06)	0.33	0.076
Shikoku	− 0.32 (− 1.32 to 0.67)	0.51	0.525
Kyushu	− 0.06 (− 0.43 to 0.31)	0.19	0.753
Period of stay in Japan (in years)			
0–4	1		
5–9	0.10 (− 0.18 to 0.39)	0.15	0.486
≥ 10	0.23 (− 0.18 to 0.64)	0.21	0.273
Formal education in Japan			
Yes	1		
No	− 0.33 (− 0.59 to − 0.06)	0.14	0.015*
Working in a Japanese company			
Yes	1		
No	− 0.04 (− 0.32 to 0.25)	0.15	0.802
Type of housing			
Weak	1		
Concrete	− 0.27 (− 0.54 to 0.01)	0.14	0.055

*SEE* Secondary education Examination, *SE* robust standard error, *CI* confidence interval.

*p-value < 0.05, **p-value < 0.01, ***p-value < 0.001.

**Figure 1:**
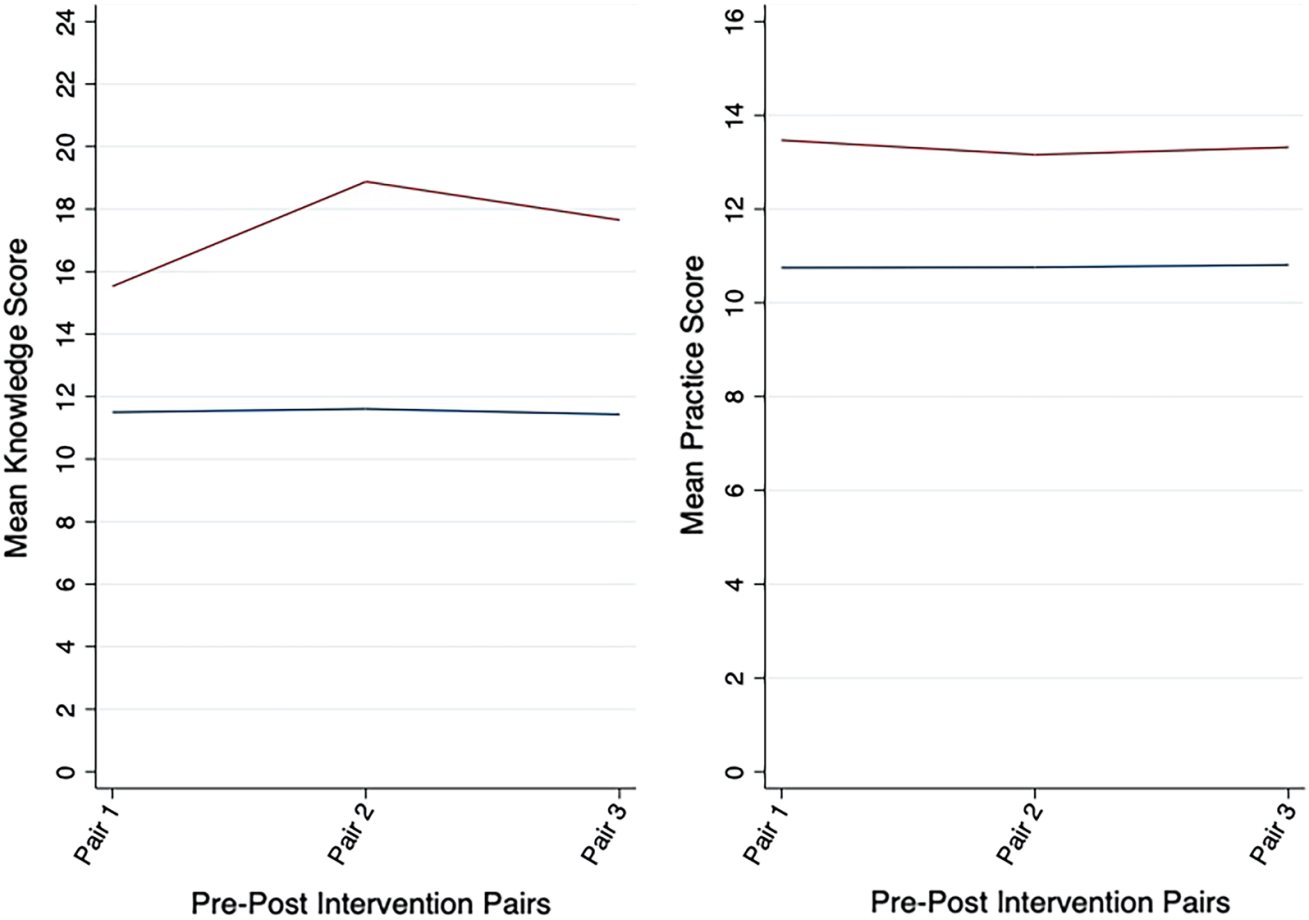
Shows the mean change in knowledge and practice score immediately after the intervention, two weeks after the intervention and 12 weeks after the intervention compared to the preintervention score where y-axis represents the knowledge and practice score in number and x-axis represents the pre-and-post intervention pairs. The blue line indicates the mean knowledge and practice score during preintervention period and red line indicates the mean knowledge and practice score at different points of intervention. Pair 1 = preintervention and immediate postintervention; pair 2 = preintervention and two weeks postintervention follow up; pair 3 = preintervention and 12 weeks post intervention follow up and p-value for each pair was obtained by paired t-test for both knowledge and practice scores which were highly significant with p < 0.001.

Compared to the preintervention scores, the mean knowledge and practice score were significantly higher in all the postintervention surveys in our paired t-test (p-value < 0.001 for all possible pairs) however, change in mean knowledge score from preintervention to post-intervention was highest during the two weeks post-intervention follow up period (change in mean score: 7.27] and then it slightly decreased at the 12 weeks follow up. In contrast, our results showed that the change in mean practice score from pre-intervention to post-intervention was lowest during the two weeks follow up period (change in mean score: 2.40) which slightly increased at the 12 weeks follow up however, the change in knowledge score was much higher than that of the change in practice scores during all post-intervention follow ups compared to that of the pre-intervention scores (Fig. 1).

Multivariable analysis based on generalized estimating equation model showed that knowledge score of the participants was 4.01 points higher (95% CI 2.78–5.24, p < 0.001) immediately after the intervention compared with that of pre-intervention level which further increased to 7.02 points higher [95% CI (5.96–8.09), p-value < 0.001] at two weeks after the intervention and 5.96 points higher [95% CI (4.78–7.14), p-value < 0.001] at 12 weeks after the intervention after adjusting for all possible confounders (Table 2).

Similar results were obtained for the practice scores. The practice score was 2.83 points higher [95% CI (2.51–3.14), p-value < 0.001] immediately after the intervention, however, unlike the knowledge score it decreased slightly at two weeks after the intervention but still was 2.62 points higher (than the pre-intervention level) at the 12 weeks after the intervention [95% CI (2.29–2.96), p-value < 0.001] after adjusting for all possible confounders. We also found some other factors which had significant association with the practice score like the province of Nepal, region of Japan, and having formal education in Japan. Compared to province 1, people from Bagmati province had practice score 0.56 points lower [95% CI (− 0.80 to − 0.09), p-value < 0.05] after adjusting for all other confounders. Similarly, participants from Tohoku region had 0.42 points higher scores [95% CI (0.08–0.76), p-value < 0.05], Chubu region had about 0.57 points higher [95% CI (0.07–1.08), p-value < 0.05], and Kansai region had 0.76 points higher [95% CI (0.19–1.34), p-value < 0.05] practice scores than those residing in the Kanto region of Japan. Meanwhile, compared to those who had formal education in Japan those who did not, had 0.33 points lower practice score [95% CI (− 0.59 to − 0.06), p-value < 0.05] after adjusting for all covariates under analysis (Table 3).

## Discussion

To the best of the authors’ knowledge, this is the first study to develop an educational intervention to enhance the earthquake preparedness knowledge and practice targeted to Nepali immigrants using their local language in Japan. Our results showed that the educational intervention provided in Nepali language was able to increase the knowledge and practice of the participants regarding earthquake preparedness. Meanwhile, several factors like place of residence in Japan and place of origin from their home country and having formal education in Japan was associated with the earthquake preparedness practice.

Engaging individuals and communities in disaster preparedness measures has been highly recommended for most disaster-prone areas^47^. However, immigrants do not tend to practice disaster preparedness measures often due to several hindrances like not having enough knowledge, language barriers, etc.^48^. The results of our study prior to the intervention also showed that there was low earthquake preparedness knowledge and practice among Nepalese immigrants residing in Japan^15,49^. This might increase the risk of morbidity and mortality among them if a high intensity earthquake occurs. Several studies have proved that there are many low-cost interventions to enhance disaster knowledge and practice among individuals like preparing emergency kits, gathering information on disaster management responses of the community, actively participating in free of cost disaster drills and practice sessions, etc.^50–52^. The educational intervention in this study also included information on earthquake preparedness, preparing emergency bags, ways of gathering information on earthquake preparedness, and so on. The intervention was provided once however, we followed up our participants in 2 and 12 weeks after the intervention and their preparedness level significantly increased in the follow up studies suggesting better retention of knowledge among the participants. Japan experienced several earthquakes in between our intervention and follow up periods which might have been one of the factors that we had higher knowledge retention rates than other studies^53^. However, integrating this educational program in a long-term earthquake preparedness program might be effective.

Many studies identified that people living in disaster prone areas are naturally prepared for disaster situations, but it was not relevant in this study^54^. People residing in the Tohoku, Kansai and Chubu region have higher earthquake preparedness practice scores than those residing in the Kanto region (a highly populated region including the several disaster prone areas like Tokyo city, Setagaya city, etc.)^55,56^. Similarly, those who had formal education in Japan had a higher practice rate than those who had not which might suggest that people who are practicing disaster drills in an educational institution or who have access to drills and practices have better earthquake preparedness. Education is considered an important tool when it comes to changing the behavior pattern of people meanwhile having formal education in Japan might imply that the participants have a good Japanese language skill which is an added advantage when it comes to preparing themselves for disaster preparedness as many official websites and smart phone applications have been introduced by Japan government to provide information on earthquake preparedness in Japanese language^57^.

Even though Japan is highly prone to earthquakes Nepalese residing in Japan had little knowledge on disaster preparedness and additionally, compared to Nepalese residing in other parts of the world like the United Kingdom where the official language is English those residing in Japan seem to have poor health seeking behavior^58^. Thus, despite of several workplace interventions like fire drills and disaster drills in Japan, working in a Japanese company did not have any change in the knowledge and practice level of our participants^56,59^. This might be due to the language barrier, higher workload or due to the poor health seeking behavior of Nepalese in general as explained by previous researches in Japan. Hence, it is crucial to implement disaster drill and practices in an organization with language assistance services otherwise in case of emergencies many immigrants might be at higher risk of death and disability. However, having a certain level of Japanese level as a mandate while applying for the residency to Japan might have a huge impact to reduce the future language related challenges among immigrant population.

This was the first intervention in Nepali language focusing on earthquake preparedness specially for the Nepalese residing in Japan and we prepared our intervention in basic Nepali language and avoided using complicated words for the clear understanding of the participants. We included information that might be relevant to Nepalese residing in any parts of Japan. Providing education or awareness on preparedness has been proven to increase the household earthquake preparedness^60,61^. Similarly, the use of social networking sites and online platforms in information dissemination has been proven to be effective by other researchers as well hence, we used online platform zoom to provide our intervention^62^. Based on prior studies we identified that the inability to participate in the disaster drills and the inability to gather information related to disaster preparedness among Nepalese immigrants is mainly due to the fact that most of Nepalese residing in Japan do not have a good Japanese language skill^15^. Nepalese coming to Japan are mainly students and blue-collar workers. Both of these groups of population have to work much more than they are legally permitted to maintain their daily living in Japan^49^. This might be the reason that they do not have enough time to participate in disaster drills. The current intervention and the follow ups were performed on the weekends during late evenings so, intervention focusing on the convenience of the target population might be effective. However, period of stay in Japan was not significant to increase the knowledge or practice in our study, people with more than 10 years of stay in Japan were not likely to increase their knowledge or practice level which might imply that these people even though have felt the devastating earthquake of 2011 in Japan did not have any motivation to seek information for or either were not able to find information related to earthquake preparedness. Hence, it is important to encourage them and provide information related to earthquake preparedness every now and then to bring change in their existing behavior and future research should focus on reducing the disparities in health care services’ accessibility and utilization in general.

This study represented the initial step in the development and validation of the tool to measure the effectiveness of an educational intervention regarding earthquake preparedness among Nepali immigrants residing in Japan. However, several limitations of the study should be considered. First, there might be some variations in the outcome measure due to the frequent earthquakes that hit Japan during the time frame of the study, such disasters might have helped in the knowledge retention process of the participants naturally. Second, this was a single-arm quasi experimental study hence, having a control group to assess the change in perceived knowledge and practice before and after intervention would have provided much more accurate estimates of the efficacy of intervention than the current one-arm interpretations similarly, the true effect of the intervention into practice could be examined by calculating survival rates during an earthquake however, we were not able to do so hence, the effectiveness of this intervention in saving lives of people during an earthquake is yet to be identified. Third, bringing change in practice was a major challenge of this study, we cannot ascertain the sustainability of the acquired practice and the knowledge retention in long term. However, we were able to publish the intervention as a booklet and distributed it among various stakeholders of Nepalese residing in Japan like Nepal Embassy, Non-Resident Nepalese Association, various societies and so on and we are planning to create an electronic version of the intervention so that people could access it from anywhere and anytime. Fourth, the participants were recruited using social networking sites like Facebook hence, it might be possible that the respondents providing us with the consent might already be a group of people who were highly motivated to learn new skills thus the intervention might not have similar impact upon other group of population however, through the web-based purposive sampling technique, the sample data used in these analyses came from a wide range of representative samples. Lastly, despite of several reminder emails there were still few significant dropouts across various categories of sex and education, this could have been prevented by more frequent reminders using some of the highly used social networking sites’ application like Facebook messenger, LINE, VIBER, etc., we believe that the equal participation of the sample across various subcategories of the socio-economic distribution would have increased the level of heterogeneity along with the generalizability of the study findings.

However, given these findings, the earthquake preparedness tool and the intervention can be considered an effective tool in enhancing the earthquake preparedness knowledge and practice among Nepalese immigrant population residing in Japan. The intervention published in the form of a booklet will hopefully encourage people to prepare ahead of time for an earthquake disaster.

## Conclusion

The current intervention can increase the earthquake preparedness knowledge perception and practice among Nepalese immigrants residing in Japan if provided at a regular interval of time. The tool developed can be used to identify the level of knowledge, attitude and practice that individuals have regarding earthquake preparedness by assessing various factors like risk perception, information seeking behavior, disaster practice measures, etc. This educational intervention could be included in various websites of the Japanese government to enhance the earthquake preparedness knowledge and practice among Nepalese immigrants residing in Japan. Similarly, this tool might be useful among the wider population of Nepalese immigrants residing in other parts of the world as it is in their native language with simpler and understandable texts. However, learning Japanese language and participating in the disaster drill sessions whenever organized by the educational institutions, work institutions or even by the government organizations of Japan might be of added advantage for those who wish to stay for longer duration in Japan for education, work or for any other reasons.

Furthermore, the intervention developed in the form of a booklet was highly appreciated by the Nepali community. Many health care professionals, experts, social workers, volunteers from various Nepalese organization including and not limited to some Japanese organizations shared their perspectives upon the earthquake preparedness booklet. Hence, it can be rest assured that the Nepali social organizations and Japanese organizations too welcomes the intervention packages which improves health care accessibility among immigrant population thus, separate trainings to the representatives of various social welfare organizations working for immigrant population might play a vital role in disseminating the education to the general people.

## Supplementary Information

Supplementary Information.

## Data Availability

The original questionnaire, educational materials and datasets used and/or analyzed during the current study are available from the corresponding author only on reasonable request to protect the privacy of the respondents as the dataset have some identifying variables of the participants and the materials used in this study are in the native language of Nepal. However, the English version of the questionnaire used has been provided as a Supplementary file (Supplementary file 1).

## Abbreviations

US: United States
EFA: Exploratory factor analysis
NRNA: Non-Residential Nepalese Association
CVR: Content validity ratio
FGD: Focus group discussion
CVI: Content validity index
I-CVI: Item level content validity index
S-CVI: Scale level content validity index
IRB: International Review Board
UK: United Kingdom
SEE: Secondary Education Examination
CI: Confidence interval
SE: Standard error
SNS: Social networking sites
GEE: Generalized estimating equation
