# Supplementary material for: Enhancing earthquake preparedness knowledge and practice among Nepalese immigrants residing in Japan
Source: Sci Rep. 2023 Mar 18;13:4468. doi: 10.1038/s41598-023-31729-y (PMC10024757; doi:10.1038/s41598-023-31729-y)
Supplement: Supplementary file 1 — Supplementary Information. [file 41598_2023_31729_MOESM1_ESM.pdf]

Supplementary file 1: Earthquake preparedness survey questionnaire

A. Earthquake preparedness general knowledge and practice related checklist (please click on either Yes/No/Not applicable or don't know for each of the following questions)

S.N.		Yes	No	Not applicable/ Don't know
1.	Have you experienced an earthquake during your stay in Japan?			
2.	Compared to other buildings in your area, do you think your house is more vulnerable to earthquake damage?			
3.	Compared to other buildings in your area, do you think your workplace is more vulnerable to earthquake damage?			
4.	Are you aware of the disaster management policies of your workplace or school?			
5.	Do you know an evacuation point near your home?			
6.	Have you ever seen a "Building Collapse Hazard Map" or a "Fire Hazard Map" for your area?			

7.	Are you familiar with earthquake mitigation measures?			
8.	Do you know where you should keep your emergency bag?			
9.	Are you familiar with the phrase, “drop, cover and hold”?			
10.	Do you know the necessary contact numbers such as fire station, police, and emergency?			
11.	Do you know how the disaster shelter works during major disasters?			
12.	Do you have a working fire extinguisher at home?			
13.	Do you know where to obtain earthquake mitigation information from?			
14.	Do you think an earthquake disaster will have an impact on your mental health?			
15.	Do you have an evacuation plan at your home and workplace?			
16.	Have you ever walked the route from your home to your evacuation point?			
17.	Did you secure your furniture like a cupboard to the wall?			
18.	Do you know how to prepare a stockpile?			

19.	Have you prepared an emergency bag including a torch, food items, first aid kit and other essential things?			
20.	Do you know how to use the fire extinguisher?			
21.	Do you practice disaster drills at your workplace or schools?			
22.	Have you discussed what to do during an earthquake among your family members?			
23.	Did you receive any mental health counseling after you were affected by the earthquake?			

B. Questions related to awareness regarding earthquake preparedness (Please click on either True/False/Don't know for each of the following statements)

S.N.		True	False	Not applicable
1.	In an earthquake you should move to open area			
2.	In an earthquake, you should get under a big piece of furniture such as a desk or other covers			
3.	You should check if your fire extinguisher or items inside the emergency back is working once a year			

4.	In an earthquake, you should hold on to a firm object until the end of the shaking			
5.	In an earthquake, you should stand near the doorway			
6.	In an earthquake you should ask your children or other family members to run to be safe from injuries during the shake			
7.	In an earthquake, if you are indoors, you must immediately exit the building			
8.	You should not use objects which have a fire hazard like gas, lighters during the earthquake.			
9.	Helping yourself during the shake should be your priority			
10.	If you are in bed during an earthquake, you should stay there and cover your head with a pillow			
11.	Once the shake has stopped you should go back to your home immediately			
12.	You should not use lifts during earthquake			
13.	In an earthquake, you should stay next to pillars of buildings and interior walls corners by lying down and covering your head			

14.	In an earthquake you should run to the higher land if you are close to sea or ocean			
15.	It is safer to have one emergency bag per person for disaster preparedness			

C. Socio-demographic characteristics related questions

1. Age (in numbers) ……………………….
2. Date of birth (in AD) ………………………
3. Sex
   i. Male
   ii. Female
   iii. Others
4. Highest education level
   i. SLC/ SEE or above
   ii. Up to +2/ high school
   iii. Bachelors or vocational training
   iv. Masters and above
5. Have you had any formal education in Japan?
   i. Yes
   ii. No
6. Residence in Nepal
   i. Province 1
   ii. Province 2

iii. Bagmati province

iv. Gandaki province

v. Lumbini province

vi. Karnali province

vii. Sudurpaschim province

7. Residence in Japan (Prefecture)

1"Hokkaidō" 2"Aomori" 3"Iwate" 4"Miyagi" 5"Akita" 6"Yamagata" 7"Fukushima" 8"Ibaraki" 9"Tochigi" 10"Gunma" 11"Saitama" 12"Chiba" 13"Tōkyō" 14"Kanagawa" 15"Niigata" 16"Toyama" 17"Ishikawa" 18"Fukui" 19"Yamanashi" 20"Nagano" 21"Gifu" 22"Shizuoka" 23"Aichi" 24"Mie" 25"Shiga" 26"Kyōto" 27"Ōsaka" 28"Hyōgo" 29"Nara" 30"Wakayama" 31"Tottori" 32"Shimane" 33"Okayama" 34"Hiroshima" 35"Yamaguchi" 36"Tokushima" 37"Kagawa" 38"Ehime" 39"Kōchi" 40"Fukuoka" 41"Saga" 42"Nagasaki" 43"Kumamoto" 44"Ōita" 45"Miyazaki" 46"Kagoshima" 47"Okinawa"

8. What kind of house do you stay in in Japan?

i. Weak house (made of wood, old homes)

ii. Concrete (mansion, earthquake resistant homes)

9. Period of stay in Japan

i. 0-4 years

ii. 5-9 years

iii. 10 years or above

10. Do you live in a neighborhood with many Nepalese?

i. Yes

ii. No

11. Do you work in a Japanese company?

I. Yes

II. No

12. Do you think online medium could be effective to deliver message on earthquake preparedness?

    Yes

    No

    If no, what do you think could be the best medium to provide educational intervention during this pandemic besides online?

    ………………………………………………………………………

13. Contact number ………………………

14. Email ……………………………….
